# Membrane potential shapes regulation of dopamine transporter trafficking at the plasma membrane

**DOI:** 10.1038/ncomms10423

**Published:** 2016-01-25

**Authors:** Ben D. Richardson, Kaustuv Saha, Danielle Krout, Elizabeth Cabrera, Bruce Felts, L. Keith Henry, Jarod Swant, Mu-Fa Zou, Amy Hauck Newman, Habibeh Khoshbouei

**Affiliations:** 1 Department of Neuroscience, Evelyn F. and William L. McKnight Brain Institute, University of Florida, Gainesville, Florida 32610, USA; 2 Department of Basic Sciences, University of North Dakota School of Medicine and Health Sciences, Grand Forks, North Dakota 58203, USA; 3 Medicinal Chemistry Section, Intramural Research Program, National Institute on Drug Abuse, Baltimore, Maryland 21224, USA

## Abstract

The dopaminergic system is essential for cognitive processes, including reward, attention and motor control. In addition to DA release and availability of synaptic DA receptors, timing and magnitude of DA neurotransmission depend on extracellular DA-level regulation by the dopamine transporter (DAT), the membrane expression and trafficking of which are highly dynamic. Data presented here from real-time TIRF (TIRFM) and confocal microscopy coupled with surface biotinylation and electrophysiology suggest that changes in the membrane potential alone, a universal yet dynamic cellular property, rapidly alter trafficking of DAT to and from the surface membrane. Broadly, these findings suggest that cell-surface DAT levels are sensitive to membrane potential changes, which can rapidly drive DAT internalization from and insertion into the cell membrane, thus having an impact on the capacity for DAT to regulate extracellular DA levels.

## Abstract (web summary)

The dopaminergic system has important roles in a number of cognitive process. Here, the authors use detailed analysis of dopamine transporter trafficking to show its levels at the cell surface are sensitive to changes in membrane potential.

## Graphical abstract

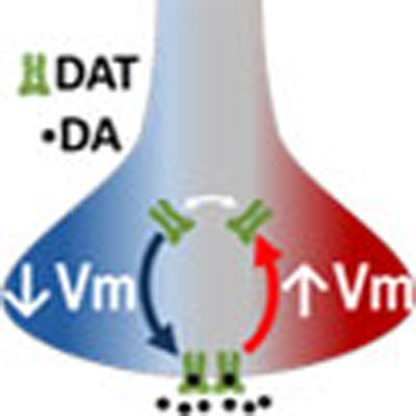

Central nervous system dopaminergic (DAergic) neurotransmission is essential in multiple neurological functions, including cognition, extrapyramidal motor control, the reward pathway and attention^1^,^2^,^3^,^4^. In addition to the timing of vesicular release of dopamine (DA) and the expression profiles of G-protein-coupled DA receptors^5^,^6^, one major regulator of DA signalling magnitude and timing is the DA transporter (DAT), which rapidly transports extracellular DA into the intracellular space for vesicular re-packaging or effluxes DA through reversal of DAT-mediated transport^7^,^8^. Commonly abused psychotropic drugs, amphetamine (AMPH), methamphetamine and cocaine achieve their effects either by inducing DA efflux through DAT and/or blocking DA uptake^9^,^10^,^11^.

The physiological function of DAT to remove DA is coupled to the translocation of one Cl^−^ and two Na^+^ ions^8^,^12^,^13^, and can even function in the absence of substrate, conducting an uncoupled, cocaine-sensitive, depolarizing current under physiological conditions^13^,^14^, which is increased in hyperpolarized states^10^. In addition to direct modulation of transport function, DAT density at the cell membrane, and therefore its functional capacity, is also dynamic. Regulated trafficking mechanisms control surface-membrane DAT levels under physiological conditions^15^,^16^ and in response to DAT substrates^15^,^16^, thus having an impact on DA homeostasis. Cell signalling molecules involved in the regulation of DAT trafficking range from protein kinase C (PKC)^17^,^18^, mitogen-activated protein kinase^19^ to Akt (ref. 20) among others^15^,^16^ and determine the presence of DAT in regulated or constitutive pools segregated to specific membrane microdomains^21^,^22^,^23^. Many DAT substrates also influence DAT trafficking^15^,^24^,^25^, including DA and AMPH, which decrease DAT surface density^26^,^27^,^28^, and cocaine, which increases DAT surface expression^29^.

Interestingly, AMPH's effects are twofold, as it causes DAT internalization^26^,^27^,^28^ and a DAT-dependent membrane depolarization^13^,^14^, which suggests an influence on DAT trafficking via a voltage-dependent mechanism in addition to DAT phosphorylation. Indeed, previous studies using striatal synaptosomes have revealed a reduction in DA uptake in depolarized (elevated KCl) conditions^30^,^31^, while *in vitro* preparations have suggested elevated DAT function at hyperpolarized states^13^. However, it is not known whether these changes in functional capacity arise from changes in ionic driving forces, essential for DA transport, changes in DAT protein presence at the membrane or both. While changes in the cell membrane voltage state are only typically considered in terms of neurotransmitter release, action potential generation and timing or in the activity of voltage-sensitive transmembrane proteins, it is possible that changes in membrane potential (MP) alone may rapidly and reversibly affect DAT trafficking to and from the cell surface. Here we use confocal and total internal reflection fluorescence microscopy (TIRFM), biochemistry, electrophysiology and optogenetics to demonstrate the degree to which surface-membrane DAT levels are shaped by and sensitive to MP changes.

## Results

### MP depolarization reduces membrane DAT levels

**Figure 1:**
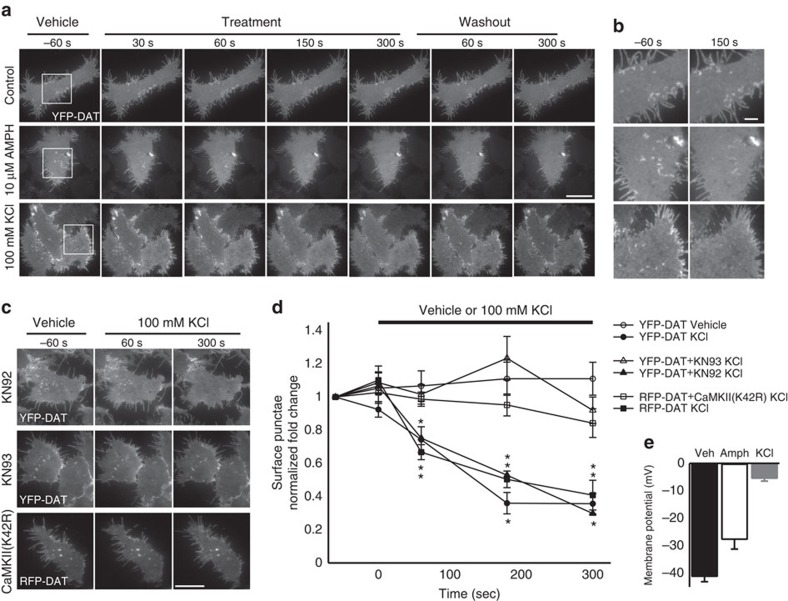
Depolarization causes CaMK-dependent reduction of membrane YFP-DAT levels in HEK cells. (**a**) Representative live cell sequential TIRF microscopy images of YFP-DAT HEK cells 60 s before and throughout 5-min perfusion with vehicle (standard external solution, top row), 10 μM amphetamine (middle row; *N*=5, *n*=14) or depolarizing 100 mM KCl-based external solution (bottom row) followed by a 5-min washout period. Scale bar, 20 μm. (**b**) Enlarged insets corresponding to boxes in left-most column before and 150 s after vehicle (top pair), 100 mM KCl (middle pair) and 10 μM amphetamine (bottom pair). Scale bar, 5 μm. (**c**) TIRF microscopy images of YFP-DAT HEK cells 60 s before and throughout 5-min perfusion, with 100 mM KCl pre-incubated with 10 μM KN92 (top), 10 μM KN93 (middle) or expressing a kinase-inactive CaMKII(K42R). Scale bar, 20 μm. (**d**) Mean±s.e.m. Normalized fold change in number of YFP-DAT puncta per cell when perfused with vehicle (open circles; *N*=5 experiments, *n*=17 cells), 100 KCl only (closed circles; *N*=6, *n*=14), 100 mM KCl when pre-incubated and perfused with 10 μM KN92 (closed triangles; *N*=5, *n*=12) or 10 μM KN93 (open triangles; *N*=6, *n*=23) and RFP-DAT puncta expressed alone (closed squares; *N*=9, *n*=9) or co-expressing a kinase-inactive GFP-CaMKII(K42R) (open squares; *N*=10, *n*=12) during 100 mM KCl perfusion. (**e**) Mean±s.e.m. Steady-state membrane potential of YFP-DAT HEK cells perfused with vehicle, 10 μM amphetamine or 100 mM KCl (*n*=5–7 cells per group). Using independent samples *t*-test *P<0.05 for comparison of 100 mM KCl effects with respective controls.

AMPH-mediated activation of DAT induces a depolarizing DAT-mediated Na^+^ current and simultaneously causes internalization of cell-surface-membrane DAT^14^,^28^. To determine whether AMPH-induced DAT internalization was the result of a psychostimulant-specific action or may be, in part, due to activation of voltage-sensitive mechanisms, we performed live cell TIRFM of yellow fluorescent protein-tagged DAT (YPF-DAT) in Human Embryonic Kidney (HEK) cells when perfused with only extracellular solution (vehicle), 10 μM AMPH or 100 mM KCl (Fig. 1), which depolarized cells by 13.5 and 35.7 mV, respectively (Fig. 1e). The distribution of YFP (yellow fluorescent protein)-DAT at the cell membrane (TIRFM footprint) was unchanged throughout perfusion of vehicle, whereas 10 μM AMPH noticeably altered the YFP signal in the TIRFM footprint within the first 60 s, causing a reduction in surface-membrane high-intensity regions and puncta that did not recover in washout (Fig. 1a,b and Supplementary Fig. 1a), in line with previous reports at longer AMPH treatment durations^32^. Similarly, depolarizing 100-mM KCl-based external solution significantly altered the YFP-DAT distribution in TIRFM footprint; however, the effects occurred rapidly, obvious within 30 s, and typically all YFP puncta and high-intensity regions were absent from the surface membrane after 3 min (Fig. 1a,b,d). In contrast to AMPH, treatment with KCl resulted in the return of YFP signal profile and the reappearance of YFP puncta immediately on washout (Fig. 1a and Supplementary Movie 1). To determine the relative specificity of this effect of depolarization for DAT, we identically depolarized HEK cells transfected with an eYFP-tagged version of an unrelated naturally occurring membrane protein, GPR40 (ref. 33), which had a membrane distribution similar to DAT, but its trafficking appeared insensitive to depolarization (Supplementary Fig. 1b,c).

Since the depolarization induced by KCl will likely increase free [Ca^2+^] and trigger the activation of Ca^2+^-dependent signalling molecules, we chose to determine the role of CaMKII and PKC in initiating this depolarization-induced redistribution. The depolarization-induced loss of YFP-DAT signal did not appear affected by the PKC inhibitor, bisindolylmaleimide I (10 μM; Supplementary Fig. 2). However, the KCl depolarization-induced loss of YFP-DAT surface puncta was significantly reduced in the presence of the CaMK inhibitor KN93 (10 μM) relative to the same treatment in the presence of the inactive homologue, KN92 (10 μM; Fig. 1c,d), which produced results similar to KCl treatment alone (Fig. 1a–d). However, because of the KN93-induced attenuation of the depolarization-triggered Ca^2+^ influx (Supplementary Fig. 3), we chose to biochemically inhibit CaMKIIα specifically and assess membrane DAT using TIRFM by co-expressing a kinase-inactive version of CaMKIIα, a green fluorescent protein (GFP)-tagged K42R mutant and RFP (red fluorescent protein)-DAT. In response to KCl-induced depolarization, RFP-DAT alone behaved similarly to YFP-DAT; however, when GFP-CaMKIIα(K42R) was co-expressed, KCl treatment was unable to alter the membrane distribution of RFP-DAT (Fig. 1c,d). These changes in membrane DAT in response to depolarization (100 mM KCl application) and repolarization (washout) suggest that the MP state is capable of bidirectionally shaping the cell-surface distribution of DAT through activation of CaMKIIα.

### Membrane DAT reduction is CaMKIIα and dynamin dependent

**Figure 2:**
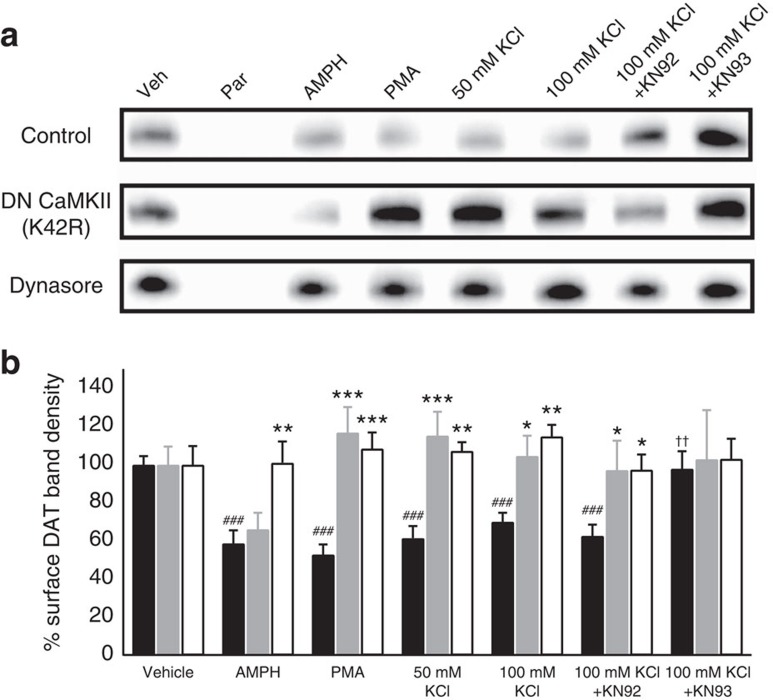
Depolarization-induced reduction in surface-membrane-biotinylated DAT is due to CaMKII-dependent endocytosis. (**a**) Representative western blots show the surface DAT present under each indicated condition. (**b**) Mean±s.e.m. DAT band density from YFP-DAT HEK cells (control, black bars) or YFP-DAT HEK cells transfected with GFP-C1-CAMKIIα-K42R (grey bars) or YFP-DAT HEK pre-treated (30 min) with external solution (vehicle) or 80 μM Dynasore (white bars), followed by 30-min treatment with vehicle, vehicle+10 μM KN92 or vehicle+10 μM KN93. Cells normalized to vehicle treatment from at least five independent experiments indicate that AMPH, PMA and depolarization-induced reduction in surface DAT are endocytosis-dependent (Dynasore-sensitive), and both PMA- and depolarization-induced internalization are CaMKIIα-dependent. Depolarization-induced internalization is sensitive to both pharmacological and molecular inhibition of CaMKII activity. A two-way analysis of variance (ANOVA) with Bonferroni *post hoc* test was performed to identify significant differences from control (*P≤0.05; **P≤0.01; ***P≤0.001), a one-way ANOVA with a Dunnett's *post hoc* test to determine changes from vehicle treatment (^###^*P*≤0.001) and an unpaired *t*-test to compare KN92 and KN93 treatments (^††^*P*≤0.01). See text for number of experiments.

To determine the degree to which real-time changes in the YFP-DAT TIRFM footprint were indicative of changes in DAT protein density at the cell membrane, a cell-surface biotinylation assay was used to quantify differences in membrane DAT protein levels. In YFP-DAT HEK cells, compared with vehicle treatment (100%, *n*=17), surface DAT (Fig. 2; see Supplementary Fig. 4 for antibody validation and total protein blots) was significantly reduced following a similar 5-min treatment as above with both 50 mM (62±6%, *n*=14) and 100 mM (70±5%, *n*=17) KCl-based external solution as well as the positive control treatments with AMPH (10 μM; 59±7%, *n*=13) and the PKC agonist phorbol myristate acetate (PMA, 2.5 μM; 53±5%, *n*=13). The CaMKIIα dependency of this effect observed in TIRFM studies was also supported by biotinylation experiments (Fig. 2), wherein 100 mM KCl had little effect on membrane DAT protein when YFP-DAT HEK cells were transfected with the kinase-inactive, dominant-negative GFP-CaMKIIα(K42R) (105±11%, *n*=6) or in the presence of the CaMK inhibitor KN93 (10 μM; 98±9%, *n*=9), whereas the inactive homologue KN92 did not significantly block the KCl-dependent reduction in surface DAT (10 μM; 63±6%, *n*=12). To determine whether the reduction in membrane DAT distribution observed using TIRFM and confirmed using biotinylation was a trafficking event, we evaluated the capacity for depolarization to induce a loss in surface DAT in the presence of Dynasore, a dynamin inhibitor^34^. Indeed, inhibition of dynamin by Dynasore blocked depolarization- (115±6%, *n*=6), AMPH- (101±11%, *n*=6) and PMA-dependent (108±8%, *n*=6) internalization of DAT in HEK cells, whereas expression of CaMKIIα(K42R) only blocked depolarization- and PMA-dependent (117±13%, *n*=6) but not AMPH-dependent (66±9%, *n*=6) internalization (Fig. 2). Importantly, treatment with 100 mM KCl did not alter surface levels of the native or overexpressed, membrane-resident transferrin receptor in comparison with vehicle control, providing further support for the specificity of depolarization-induced downregulation of membrane DAT (Supplementary Fig. 5). Taken together, these data suggest that CaMKIIα- and dynamin-dependent pathways are involved in depolarization-dependent DAT trafficking at the cell membrane.

### MP depolarization internalizes JHC 1-064/DAT complexes

**Figure 3:**
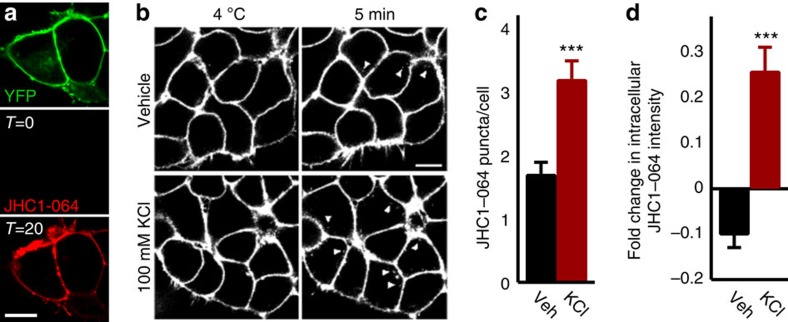
Membrane depolarization with KCl increases internalization of JHC 1-064/DAT complexes in HEK cells. (**a**) JHC 1-064 binds to surface DAT in HEK cells. Confocal image of YFP signal in YFP-DAT HEK cells (top), with no initial detectable fluorescence emitted between 553 and 617 nm when excited with 561 nm (bottom left, T=0 min) until after 20-min exposure to 10-nM JHC 1-064 (bottom right) corresponding with YFP fluorescence. (**b**) Representative confocal images of JHC 1-064/DAT labelling (white) at ∼4 °C and 5 min following solution change at 37 °C. Note the increase at 5 min in the number of white intracellular puncta in the KCl condition. (**c**,**d**) Mean±s.e.m. number of individual JHC 1-064/DAT puncta per cell (**c**) and normalized (to 4 °C) fluorescence intensity (**d**) in the intracellular space corresponding to vehicle (*N*=3 experiments, *n*=69 cells, black bars) or 100 mM KCl-containing solution treatment (*N*=3 experiments, *n*=85 cells, red bars). Independent samples *t*-tests were used to compare vehicle with KCl effects. Scale bars, 10 μm. ***P<0.0001 using independent samples *t*-test.

Next, we used JHC 1-064 (ref. 35), a fluorescent cocaine analogue, to label cell membrane-resident DAT in HEK FLAG-DAT cells in conjunction with live cell confocal microscopy to investigate whether KCl-induced membrane depolarization would drive the internalization of cell-surface JHC 1-064/DAT complexes, an approach used previously to study DAT trafficking to defined endosome compartments *in vitro* (Fig. 3a)^36^. In all cases, the presence of JHC 1-064/DAT complex puncta in the intracellular space was limited or nonexistent at 4 °C (Fig. 3b). However, when changing bath temperature from 4 to 37 °C with either vehicle or depolarizing (iso-osmotic) 100-mM KCl-based external solution (Fig. 3b–d), the number of fluorescent punctate JHC 1-064/DAT complexes (Fig. 3c) and average intracellular fluorescence intensity (Fig. 3d) were significantly greater in depolarizing KCl-based external solution (3.2±0.3 puncta per cell; normalized fold change in intracellular JHC 1-064 intensity: 0.26±0.05) compared with vehicle (1.7±0.2 puncta per cell; normalized fold change in intracellular JHC 1-064 intensity: −0.10±0.05).

### MP depolarization redistributes DAT into early endosomes

**Figure 4:**
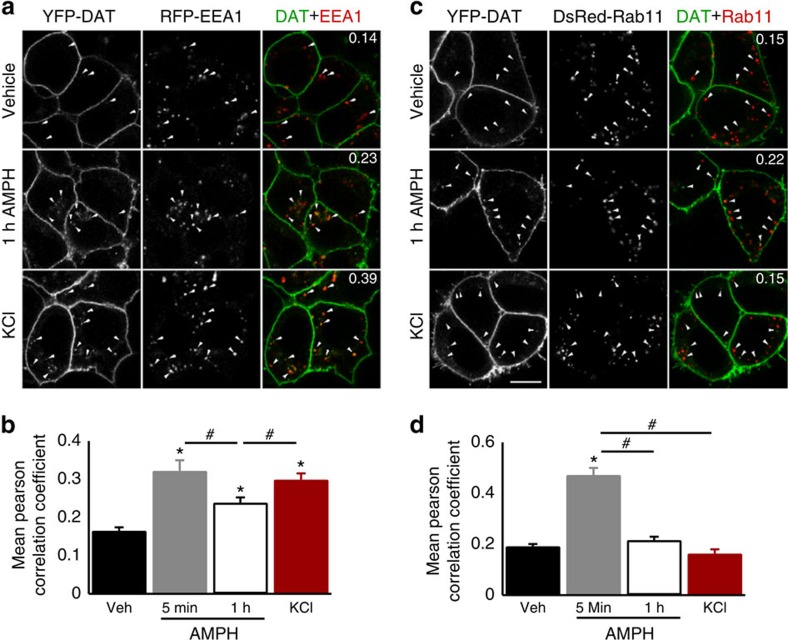
Depolarization-dependent internalized DAT preferentially localizes to early but not recycling endosomes. (**a**,**c**) Representative single-plane confocal images of (left) YFP-DAT, (middle) TagRFP-T-EEA1 (**a**) or DsRed-Rab11 (**c**) and merge of the two (right) expressed in HEK cells treated with vehicle (top; EEA1: *N*=5, *n*=106; Rab11: *N*=6, *n*=77), 10 μM amphetamine for 1 h (middle; EEA1: *N*=3, *n*=50; Rab11: *N*=3, *n*=61) or 5 min (EEA1: *N*=2, *n*=27; Rab11: *N*=2, *n*=13) or 100 mM KCl for 5 min (bottom; EEA1: *N*=3, *n*=84; Rab11: *N*=3, *n*=4). (**b**,**d**) Mean±s.e.m. Pearson correlation coefficient per cell of intracellular YFP-DAT signal with EEA1 (**b**) or Rab11 (**d**) for vehicle, 5 min and 1 h amphetamine and 100 mM KCl-treated cells. Value inset in merged images is the mean Pearsons correlation coefficient for cells in given image. Scale bar, 10 μm and applies to all images. *P<0.05 compared with vehicle, #*P*<0.05 for indicated comparison using ANOVA with Bonferroni *post hoc* test.

To determine the identity of the intracellular destination of depolarization-dependent internalized DAT, fluorescent versions of the endosome markers (which were not apparent at the cell membrane in TIRFM; see Supplementary Fig. 6), EEA1 (TagRFP-T-EEA1) that marks early endosomes or the recycling endosome marker Rab11 (DsRed-Rab11), were expressed in HEK YFP-DAT cells and then treated with standard external solution (vehicle), iso-osmotic 100-mM KCl-based external solution for 5 min or 10 μM AMPH for 5 min as a temporal comparison. Another time point of 60 min AMPH (10μM) treatment was used as a positive control as it has been shown previously to cause DAT internalization to specific endosomes^18^,^37^,^38^,^39^, and would thus allow for comparability to previous work. Average Pearson correlation coefficients per cell for intracellular YFP-DAT and TagRFP-T-EEA1 or DsRed-Rab11 in vehicle (EEA1: 0.17±0.01; Rab11: 0.19±0.01) were significantly less than in cells treated with AMPH at 5 min for EEA1 (0.32±0.03; Fig. 4a,b) and Rab11 (0.47±0.06; Fig. 4c,d), while 60-min AMPH treatment increased YFP-DAT association with EEA1 (0.24±0.01; Fig. 4a,b), but not Rab11 (0.22±0.15; Fig. 4c,d). Similarly, depolarizing conditions significantly enhanced the co-localization of intracellular YFP-DAT with EEA1 (0.30±0.02; Fig. 4a,b) over vehicle and 60-min AMPH treatments, although comparable to the 5-min AMPH treatment. The treatment had no effect on the degree of association of intracellular YFP-DAT with Rab11 (0.16±0.07; Fig. 4c,d) compared with vehicle. While biotinylation and confocal imaging inherently lack the temporal resolution of TIRFM, the collective results indicate that MP depolarization rapidly reduces cell-surface-membrane DAT and internalizes the transporter to intracellular early endosome compartments, suggesting that membrane DAT levels and DAT trafficking may be partially dependent on the MP state and therefore could change rapidly with MP fluctuation on local changes in the activity of receptors, ion transporters and channels.

### Change in MP state alters cell surface membrane DAT levels

**Figure 5:**
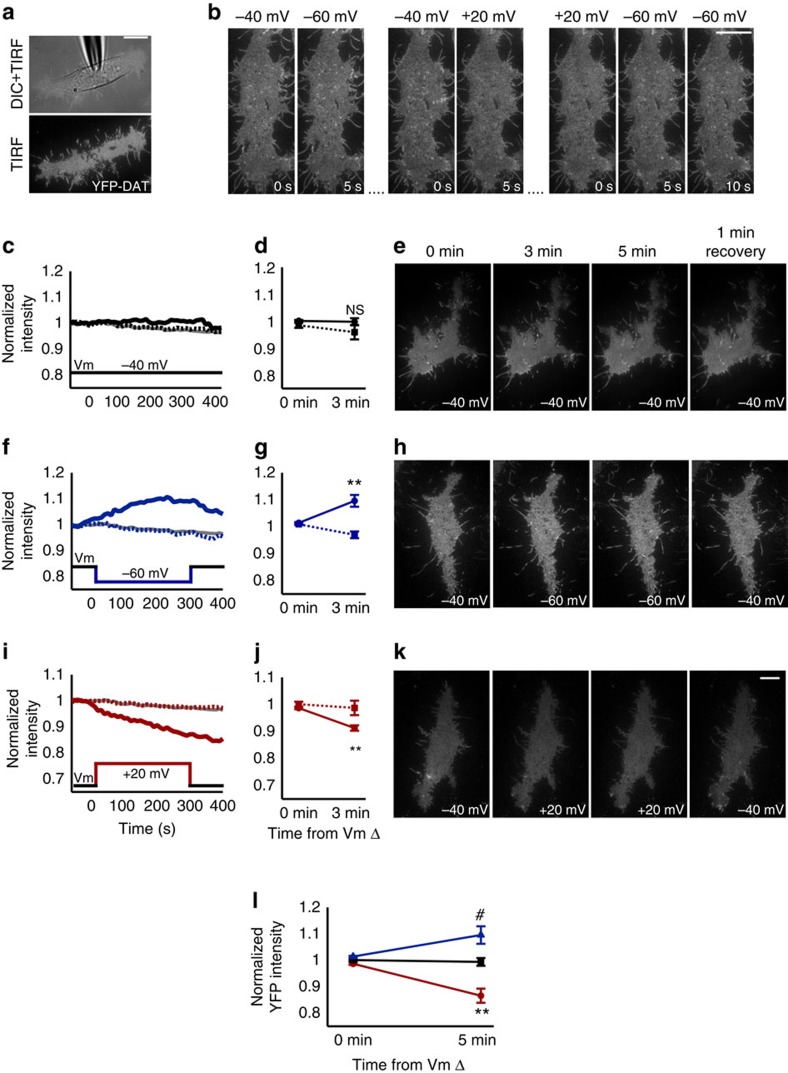
Membrane potential state alters cell membrane YFP-DAT levels. (**a**) Simultaneous whole-cell patch-clamp recording with the patch pipette visible in dissolved inorganic carbon (top) of YFP-DAT HEK cell and TIRF imaging of YFP-DAT (white) of the same cell (bottom). (**b**) TIRF image sequence with 5-s interframe interval of YFP-DAT HEK cell footprint with the membrane potential clamped at indicated holding potentials. Hyperpolarized potentials (−40 to −60 mV transition) increase cell-surface YFP-DAT signal, while depolarized potentials (−40 to +20 mV transition) reduces cell-surface YFP-DAT within 5 s and recovers within 5–10 s on hyperpolarization (+20 to −60 mV transition). (**c**,**f**,**i**) The mean normalized intensity of the surface-membrane YFP-DAT over time for all clamped cells (solid line) held at −40 mV (**c**), −60 mV (**f**) and +20 mV (**i**) for 5 min. Error bars are omitted for clarity, and the mean intensity for matched corresponding adjacent non-patched cells (colour-matched dashed line) and the mean of all non-patched adjacent cells (grey line) are included for comparison. (**d**,**g**,**j**) Normalized fluorescence intensity values for patched-clamped (solid line) and paired adjacent cells (dotted line) before (0 min) and 3 min after membrane potential change (*n*=4–5 cells per group). (**e**,**h**,**k**) Representative images of YFP-DAT HEK TIRFM footprints of patched cells before (0 min), 3 and 5 min after the membrane potential change and 1 min after returning the membrane potential to −40 mV. (**l**) Average normalized YFP intensities at 5 min after the potential change to hyperpolarized (−60 mV, blue) or depolarized (+20 mV, red) membrane potentials are significantly different relative to continuous clamping near the endogenous (−40 mV, black) membrane potential. #*P*<0.05, **P<0.001 using independent samples *t*-test. All values are mean±s.e.m. Scale bars, 10 μm.

To further examine whether the membrane distribution of DAT is altered in response to MP changes (depolarization and hyperpolarization), we employed simultaneous single-cell TIRFM and whole-cell patch clamp electrophysiology (Fig. 5a). This technique allowed for time-resolved, bidirectional and precise control of the MP and provided internal controls in adjacent non-clamped cells. Acquisition of TIRFM image sequences (5-s intervals) throughout the course of 5-min duration voltage steps indicated that the surface YFP-DAT signal is stable during whole-cell voltage clamp at −40 mV (typical MP for YFP-DAT HEK cells; Fig. 5b–e), but MP changes from baseline to hyperpolarized (−60 mV; Fig. 5b,f–h) or depolarized (+20 mV; Fig. 5b,i–k) potentials could rapidly (between frames, 5 s duration) increase or reduce, respectively, YFP-DAT puncta in the TIRFM footprint (Fig. 5b). In some cases, the 5-s interframe interval during depolarization was sufficient to eliminate all DAT puncta from the cell surface, and hyperpolarization to −60 mV caused complete recovery of the fluorescent signal profile within 5–10 s (Fig. 5b). The effect of the hyperpolarizing voltage step on the TIRFM footprint intensity of patch-clamped cells rapidly increased, typically plateauing within 5 min, and began to reverse (decrease in intensity) following return of the membrane voltage to −40 mV (Fig. 5f). Continuous clamping of the MP at −40 mV did not significantly alter the YFP-DAT TIRFM footprint intensity at 3 min relative to adjacent cells (*n*=4 clamped, four adjacent cells; *P*>0.05; Fig. 5c–e). However, when comparing intensity changes between clamped and adjacent cells 3 min into the voltage step, stepping the MP to −60 mV significantly increased the YFP-DAT TIRFM footprint intensity (*n*=5 clamped, five adjacent cells; *P*<0.01; Fig. 5f–h), while stepping to +20 mV produced the opposite effect (*n*=5 clamped, five adjacent cells; *P*<0.01; Fig. 5i–k). Further comparison of voltage effects between only cells clamped at −40, −60 or +20 mV also indicates a significant difference in normalized YFP-DAT TIRFM footprint intensity for the −60 mV (*n*=5 cells; *P*<0.05) and +20 mV (*n*=5 cells; *P*<0.001) condition when compared with the −40 mV (*n*=4 cells) condition 5 min following the voltage change (Fig. 5l).

### Change in MP state alters DAT-mediated current

**Figure 6:**
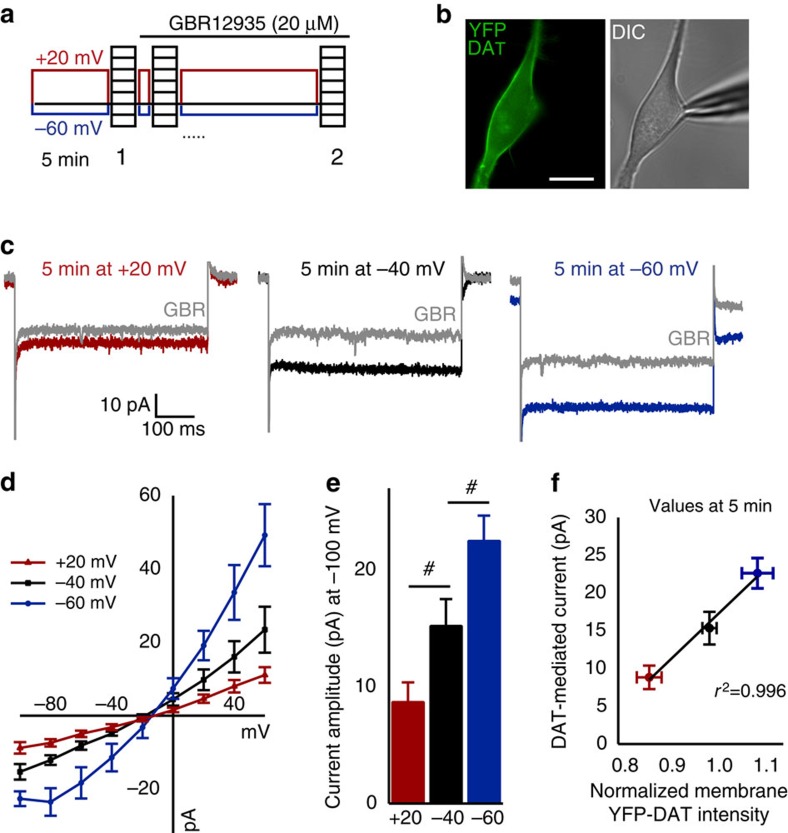
Membrane potential state significantly alters DAT-mediated current. (**a**) Schematic of the whole-cell voltage protocol for examining membrane potential state-dependent changes in DAT-mediated current recorded from HEK cells expressing YFP-DAT (see Methods). (**b**) Representative image of an YFP-DAT HEK cell following the complete recording paradigm for the +20-mV condition. Scale bar, 10 μm. (**c**) Representative current responses of the −100-mV voltage step before (coloured trace) and after 20-μM GBR12935 (grey trace) for the given prolonged holding potential condition. (**d**) Group average IV plots for each voltage-state condition. (**e**) Bar graphs depict the mean current amplitude at −100 mV step from full IV plot for each condition and were used for statistical comparison between groups (*n*=5–8 cells per group). (**f**) Average DAT-mediated current amplitude plotted against the relative mean fraction of original intensity of the YFP-DAT TIRFM footprint (from Fig. 5) for cells patched at control, hyperpolarized and depolarized potentials indicates a positive correlation between increasing DAT-mediated current density and peri-membrane YFP intensity with TIRF microscopy. ^#^*P*<0.05 using independent samples *t*-test. All values are mean±s.e.m.

Since the mere presence of DAT at the cell surface (YFP fluorescence signal) is not necessarily indicative of relative DAT function, we sought to determine whether MP change-induced variations in the surface DAT density (Fig. 5) correlated with the uncoupled DAT-mediated current. To investigate this, as in previous TIRFM experiments, the MP was clamped for 5 min at +20, −40 or −60 mV but was followed by acquisition of a baseline IV curve (Fig. 6a). The subsequent GBR12935-sensitive current was then taken as the DAT-mediated current for each cell for a given condition. Cells clamped at −40 mV (near their endogenous resting MP) had a DAT-mediated current amplitude of −15.3±2.16 pA (*n*=8; Fig. 6c–f, black). However, when cells were depolarized to +20 mV for 5 min (Fig. 6c–f, red), the DAT-mediated current (−8.8±1.6 pA, *n*=7) was significantly reduced by 42.3% (*P*<0.05), and cells hyperpolarized to −60 mV for 5 min (Fig. 6c–f, blue) displayed a 47.7% larger (*P*<0.05) DAT-mediated current (−22.6±2.0 pA, *n*=5) compared with cells held at −40 mV. For comparison of MP state-dependent changes in YFP-DAT membrane density (Fig. 5) and DAT functional capacity, the average fold change in YFP-DAT TIRFM footprint intensity and DAT-mediated current amplitude for each MP-holding potential state are plotted against each other (Fig. 6f), indicating a positive correlation between the two measures. While the cell-surface-membrane DAT levels (TIRFM) are influenced by the MP state, these data imply that functional DAT may be particularly influenced by MP state changes as they are more profoundly affected.

### Neuronal MP changes alter surface-membrane DAT levels

**Figure 7:**
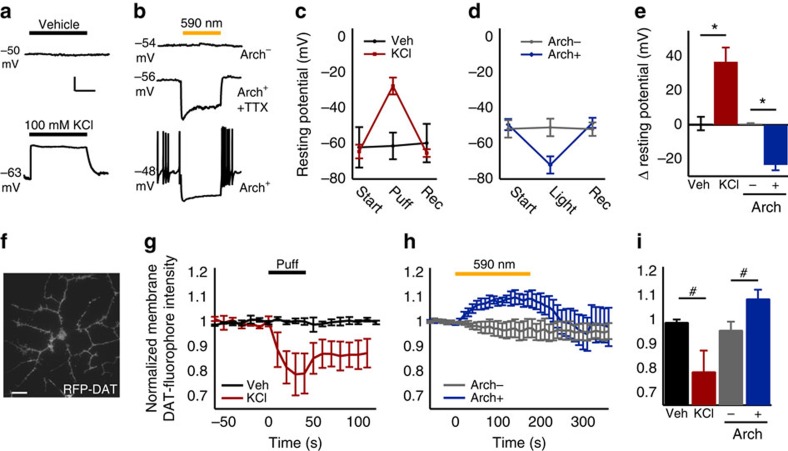
Neuronal membrane potential rapidly alters membrane-surface DAT level. (**a**,**b**) Cultured neuron membrane potential response to (**a**) vehicle or 100 mM KCl and (**b**) 590 nm stimulation in neurons with/without YFP-Arch 0.5 mM TTX omitted in bottom trace. Scale bar, 10 s (**a**) and 500 ms (**b**) and 10 mV for (**a**,**b**). (**c**) Membrane potential before, during and after vehicle (black; *n*=3) or 100 mM KCl (red; *n*=4) and (**d**) 590-nm light stimulation of neurons with (blue; *n*=13)/without (grey; *n*=3) Arch-YFP expression. (**e**) Resting potential change. (**f**) TIRFM image of primary culture neuron expressing RFP-DAT. (**g**) The mean normalized intensity of the surface RFP-DAT during vehicle (black; *n*=4) or 100 mM KCl (red; *n*=3) application. (**h**) The mean normalized intensity of the surface CFP-DAT when a 590-nm light pulse was delivered to cells with (blue; *n*=5)/without (grey; *n*=4) Arch-YFP expression. (**i**) Normalized somatic membrane RFP- or CFP-DAT fluorescence change for each condition. ^#^*P*<0.05, *P<0.01 with independent samples *t*-test.

To determine whether MP influences surface-membrane DAT density in functional neurons, real-time imaging of membrane DAT (TIRFM fluorescence footprint) was coupled with two characterized non-invasive methods (Supplementary Fig. 7) of transient reliable membrane depolarization and hyperpolarization: 100-mM KCl focal application (Fig. 7a) and archaerhodopsin (Arch) activation (Fig. 7b), respectively. Cultured primary neurons were transfected with TagRFP-T-DAT (RFP-DAT) or CFP-DAT with or without co-transfection with Arch-YFP (Arch-YFP; Supplementary Fig. 7) and were subjected to whole-cell recordings (K-gluconate-based internal solution) or imaging in the presence of tetrodotoxin (TTX) and receptor blocker cocktail (see Methods), unless otherwise indicated. Pressure application of KCl-based external solution induced a reversible 36.9±8.3-mV membrane depolarization in RFP-DAT-expressing neurons (Fig. 7a,c–e), and photo-activation (590 nm) of Arch caused a reversible −23.3±3.2-mV membrane hyperpolarization (Fig. 7a,c–e). In the absence of TTX, Arch activation suppressed action potential firing and induced a rebound burst when the light was turned off (Fig. 7b) and was relatively stable over long pulse durations (Supplementary Fig. 7) used for subsequent imaging experiments^40^. The MP of neurons lacking Arch-YFP expression was unaffected by 590-nm light stimulation.

On characterizing the reliability of these tools, we performed simultaneous TIRFM of primary culture neurons (Fig. 7f) during each manipulation (Supplementary Fig. 7b and Fig. 7g,h). TIRFM of neurons expressing RFP-DAT while focally applying 100-mM KCl-based external solution (Supplementary Fig. 7b) for a short duration (45 s) caused a rapid and dramatic reduction (−21.1±8.5%) in the RFP-DAT TIRFM footprint intensity (Supplementary Movie 2), while vehicle application had no effect (Fig. 7g). Similarly, bath application of 100-mM KCl-based external solution also enhanced the internalization of endogenous DAT in primary neurons labelled with JHC 1-064, causing a dramatic increase in JHC 1-064 complexes in the intracellular space (Supplementary Fig. 8). In contrast, simultaneous MP hyperpolarization via activation of Arch by 590-nm light staggered with TIRFM imaging of CFP-DAT (Fig. 7h) indicated that hyperpolarization caused a reversible increase (+9.0±3.5%) in CFP-DAT intensity in the TIRFM footprint overtime, which stabilized after 120 s (Fig. 7h and Supplementary Movie 3). No significant change (−3.8±3.3%) in CFP-DAT TIRFM footprint intensity was observed in neurons lacking Arch-YFP expression (Fig. 7h). These KCl-induced decreases and Arch activation-induced increases in neuronal cell-surface DAT TIRFM signal, which parallel MP state-dependent changes in the surface density of DAT protein (Figs 1, 2, 3, 4, 5) and DAT function (Fig. 6) in YFP-DAT HEK cells, were significantly different from their respective controls (depolarization: *P*<0.05, hyperpolarization: *P*<0.05; Fig. 7i).

## Discussion

The presence of DAT at the cell membrane is crucial in the regulation of DAergic signalling, timing and magnitude throughout the brain, and thus any alteration in the functional capacity of the transporter may significantly have an impact on neurological functions in which DA is involved. Previous studies have demonstrated that KCl-induced depolarization reduces DA uptake^30^,^31^, and that membrane hyperpolarization increases DAT-mediated inward current and DA uptake^13^, albeit with an unknown mechanism. Here we asked whether changes in MP alone may rapidly and reversibly regulate DAT trafficking. One aspect regulating transporter function is that the trafficking of mature DAT to and from the cell membrane is a highly regulated process, which is affected in various disease states and by the activity of DAT-targeting psychostimulants. Using live cell TIRFM and biotinylation on identically treated HEK cells expressing YFP-DAT, we determined that membrane depolarization alone could induce a CaMKIIα- and dynamin-dependent (Figs 1 and 2) rapid reversible (increase in hyperpolarization recovery) reduction in membrane DAT (Fig. 1 and Supplementary Movie 1). This depolarization-induced effect on DAT distribution in the TIRFM footprint was distinctly different when compared with the effects of AMPH, which did not recover as quickly. Another difference between AMPH- and depolarization-induced DAT internalization is the insensitivity of AMPH-induced internalization to the loss of CaMKIIα activity through the coexpression of a dominant-negative, kinase-inactive CaMKIIα (Fig. 2), which, along with the sensitivity of both versions to KN93, may suggest that different isoforms of the kinase may have distinctly different roles in regard to regulating DAT function. Notably, similar fast changes in membrane DAT levels have been reported using this approach with acute AMPH exposure^41^. However, the direction of the AMPH effect on human DAT using the multifaceted approach reported here contrasts with this previous finding and could be due to intrinsic differences between rat and human DAT, AMPH concentrations and/or cell types.

To determine the degree of DAT internalization, with the DAT-specific fluorescent cocaine analogue, JHC 1-064 (ref. 36), we followed the distribution of JHC 1-064 fluorescence (JHC 1-064/DAT complexes; Fig. 3) when cells were left at rest or depolarized. These data suggested that indeed membrane-resident DAT was being more rapidly brought into the intracellular space when depolarized as compared with constitutive internalization (Fig. 3c,d). While biotinylation and confocal imaging inherently lack the temporal resolution of TIRFM, together, results indicate that in contrast to the effects of AMPH on DAT, depolarization resulted in DAT being segregated specifically into early endosome compartments (EEA1), but not recycling endosomes (Rab11; Fig. 4). This divergence in the destination of internalized DAT in cells treated with AMPH versus those simply depolarized again suggests the involvement of differing mechanisms, which may leave initially internalized DAT residing in early endosomes free to transition into rapid recycling endosomes, distinct from recycling endosomes^42^, putatively underlying the faster recovery to the membrane surface during hyperpolarization.

Although few studies have examined DAT activity immediately after depolarization or following the return to the resting hyperpolarized state, our data provide a potential mechanism for the decreased DA uptake in striatal synaptosomes during the fast phase of depolarization-induced DA release^31^. Therefore, to determine any bidirectionality of the KCl effect on DAT trafficking, we used whole-cell voltage-clamp techniques to clamp the MP of YFP-DAT HEK cells while performing TIRFM simultaneously (Fig. 5). Once cells were clamped near their endogenous resting potential (−40 mV), the YFP-DAT TIRFM footprint was relatively similar over time (Fig. 5c–e). However, when stepping the membrane-holding potential from −40 mV to a hyperpolarized potential, an increase in YFP-DAT intensity and puncta number in the TIRFM footprint began immediately (Fig. 5b) and plateaued after 3 min (Fig. 5f–h). In contrast, when cells were depolarized the opposite effect occurred with a loss of YFP-DAT signal, which paralleled the effects seen in the presence of depolarizing KCl (Fig. 5i–k). In fact, the ∼10% change in YFP-DAT intensity directly corresponded to reductions in DAT-mediated (GBR12935-sensitive) current when cells were clamped at depolarized or hyperpolarized potentials (Fig. 6). On the basis of these data and the known electrogenic nature of DAT-mediated DA uptake and efflux, we hypothesize that at depolarized conditions, where efflux is more likely to occur^10^, the cell may actively attenuate this efflux by downregulating DAT at the membrane. In contrast, membrane hyperpolarization, known to facilitate DA uptake^13^, may be doing so through interactions with ionic driving forces and increases in membrane DAT.

With two tools that induced reversible and reliable depolarization (focal KCl application) or hyperpolarization (Arch activation) of magnitudes, similar to those used in previous experiments (Fig. 7a–e), we used TIRFM to monitor fluorescent-tagged DAT expressed in midbrain primary cultures during MP manipulation. The effect of these manipulations on membrane DAT levels were larger than in HEK cells using methods that induced similar voltage differences, implying that these effects are indeed applicable to neuronal populations and results obtained using HEK cells are relevant to shaping conclusions about MP-dependent trafficking of DAT in the nervous system. Together, these data indicate that, while the magnitude of change in membrane DAT levels due to MP changes varies depending on the assay, all changes observed are in a physiologically relevant range and the direction of the effect (increase or decrease) is in agreement across all examinations.

This effect of the MP on DAT trafficking sheds light on an additional mechanism by which the activation of hyperpolarizing D_2_Rs may be altering DA transport. The activation of D_2_Rs has long been understood to enhance DAT function^43^, and previous studies have suggested that a D_2_R activation initiates a signalling cascade to upregulate cell-surface DAT^19^. Although others have examined how changes in the neuronal MP similarly to those initiated by D_2_R activation may alter DAT function and found no impact on [^3^H]-DA uptake^44^, those experiments were performed at room temperature, which likely attenuates trafficking rates as opposed to the studies here conducted at near-physiological temperatures (37 °C). Thus, this methodological difference may explain the discrepancies between that [^3^H]-DA uptake study^44^ relative to the data presented here. Nevertheless, collectively this study and previous studies support the involvement of multifaceted regulatory mechanisms for DAT trafficking that are substrate-, kinase- and activity-dependent. The existence of multiple regulatory mechanisms supports the notion that the DAT proteins at the membrane are responsive to diverse regulatory mechanisms. The overriding mechanism for activation of a given trafficking pathway will be determined by the nature of the stimulation and the availability of specific regulatory constituents.

DA signalling is crucial in many neurological functions, as aberrations in DA neurotransmission contribute to multiple neuropsychiatric disorders, including addiction^3^,^45^, Parkinson's disease and movement disorders^46^,^47^,^48^, schizophrenia^49^,^50^ and attention-deficit hyperactivity disorder (ADHD)^2^, all of which have been linked to how extracellular DA may be mishandled by altered DAT expression and function^23^,^50^,^51^. As a result, disease-related deviations from physiological states and variations in neuronal MPs may be altering the functional capacity of DAT by affecting its trafficking to and from the membrane. This dynamic balance of electrophysiological and biochemical processes to regulate subtle but essential aspects of neurotransmission opens a range of possibilities for exploring related aberrations in disease states and in pharmacotherapy targeting this interaction. Broadly, the regulation of protein (DAT) trafficking by the MP may provide additional means by which plasticity (for example, activity-dependent changes) in DAergic and possibly non-DAergic systems is maintained and controlled.

## Methods

### Cell culture

*Cell lines*. HEK cells overexpressing FLAG-tagged or YFP-tagged human DAT (hDAT), HEK FLAG-DAT (refs 52, 53) or YFP-DAT HEK (ref. 54), respectively, were a generous gift from Dr Jonathan Javitch (Colombia University) prepared from HEK293 EM4 as previously described^55^,^56^. The addition of the YFP tag and FLAG epitope to hDAT is a widely used construct and has not been shown to alter basic functional properties of the transporter or other transporter-mediated activity^24^,^52^,^53^,^55^,^57^. HEK cells were maintained in DMEM supplemented with 10% fetal bovine serum (FBS), 5% L-glutamine, penicillin (50 μl ml^−1^) and streptomycin (50 μl ml^−1^) at 37 °C and 5% CO_2_. Cells were typically passaged and/or used for electrophysiology or imaging experiments after reaching 60–80% of full confluency (every 2–3 days). To induce expression of constructs not stably expressed in HEK cell lines, HEK293 EM4 cells were transfected using a standard calcium phosphate protocol. Transfected cells were used in experiments 12–36 h after transfection.

*Midbrain primary neuron culture*. All animals used were housed in the University of Florida's McKnight Brain Institute animal care facility, an Association for Assessment and Accreditation of Laboratory Animal Care International-accredited facility. The University of Florida's Institutional Animal Care and Use Committee approved of all procedures undertaken. Primary cultures of the ventral midbrain containing DAergic neurons were prepared as previously described^9^,^57^,^58^ and are described here in brief. The ventral midbrain, including DA neuron-rich regions, was acutely dissociated and isolated under sterile conditions from 0- to 2-day-old C57Bl/6J mice of both sexes and incubated at 34–36 °C under continuous oxygenation for 30 min in a dissociation medium, containing (in mM): 116 NaCl, 5.4 KCl, 26 NaHCO_3_, 25 glucose, 2 NaH_2_PO_4_, 1 MgSO_4_, 1.3 cysteine, 0.5 EDTA, 0.5 kynurenic acid and containing 20 units ml^−1^ papain. Subsequently, tissue was triturated with fire-polished Pasteur pipettes in glial medium, containing (in %): 50 MEM, 38.5 FBS, 7.7 penicillin/streptomycin, 2.9 D-glucose (45%) and 0.9 glutamine (200 mM). Dissociated cells were pelleted at 450*g* for 10 min and were re-suspended in glial medium. Cells were plated on 12-mm round coverslips placed in 35 × 10 mm style tissue culture Petri dishes or glass-bottom 35 × 10 mm (MatTek, Ashland, MA) coated with 100 μg ml^−1^ poly-D-lysine and 5 μg ml^−1^ laminin. One hour after plating, the medium was changed to neuronal medium, containing (in %): 2 MEM, 75 Ham's-F12 medium, 19 heat-inactivated horse serum, 2 FBS, 1.56 D-glucose (45%), 0.04 insulin (0.025 g ml^−1^) and 0.4 apotransferrin (50 mg ml^−1^). Neuronal cultures were transfected with TAGRFP-T-DAT, CFP-DAT and/or Arch-YFP constructs via nucleofection (Mouse Nucleofector Kit, programme O-005, Lonza Group Ltd, Basel, Switzerland) immediately before plating or via calcium phosphate 3–5 days after plating using standard protocols. All cultures were used at 7–9 days *in vitro* (DIV) and 4–9 days post transfection. No difference between transfection methodologies was observed regarding protein expression level and/or function.

### Plasmid constructs

The plasmid coding for the cyan fluorescent protein-tagged DAT was described previously^59^,^60^ and was provided as a generous gift from Dr Alexander Sorkin (University of Pittsburgh). The RFP-tagged hDAT (TagRFP-T-DAT)^34^, generated as previously described, was a gift from Dr Haley Melikian (University of Massachusetts), and the construct for fatty-acid receptor GPR40-eYFP driven by the cytomegalovirus promoter was a gift from Dr Sergei Zolotukhin and Seth Currlin (University of Florida). DsRed-Rab11 WT^61^, a recycling endosome marker, was a gift from Richard Pagano (Mayo Clinic and Foundation, Addgene plasmid #12679). TagRFP-T-EEA1 (ref. 62), an early endosome marker, was provided by Silvia Corvera (University of Massachusetts Medical School, Addgene plasmid #42635). The GFP-C1-CAMKIIα-K42R (ref. 63) was a gift from Tobias Meyer (Stanford University, Addgene plasmid #21221), and the pTfR-PAmCherry1 (ref. 64) plasmid was a gift from Vladislav Verkhusha (Albert Einstein College of Medicine, Addgene plasmid #31948). To confer optical control of MP hyperpolarization, neuronal cultures were transfected with AAV-CaMKα-eArch 3.0-EYFP plasmid, a generous gift from Dr Karl Deisseroth (Stanford University). Arch was chosen for its ability to induce large magnitude H^+^-current-hyperpolarizing shifts (10–50 mV) in the neuronal MP, which were relatively stable over seconds to minutes with minimal decay when continuously activated^40^,^65^.

### Electrophysiology

HEK cells and cultured neurons were visualized with a × 60 objective on an inverted Nikon Ti Eclipse microscope (Nikon, Melville, NY). All currents and MPs were recorded via an Axoclamp 200A amplifier using the whole-cell configuration after forming a high-resistance seal in the cell-attached configuration (>1 MΩ). All signals were digitized with a Digidata 1440A at 10 kHz, and a 5-kHz low-pass Bessel filter was applied during acquisition. An additional 2-kHz Gaussian filter was applied to all traces for presentation only. The standard external solution for electrophysiology experiments using HEK cells was the same used in all microscopy and biochemistry experiments and contained (in mM) the following: 130 NaCl, 10 HEPES, 34 Dextrose, 1.5 CaCl_2_, 0.5 MgSO_4_ and 1.3 KH_2_PO_4_, with a pH of 7.35 and osmolarity of 275–290 mOsm. Pipettes for whole-cell recordings were pulled from borosilicate glass on a P-2000 laser-based puller (Sutter Instruments, Novato, CA). Pipettes used for recording the MP (3–6 MΩ) were filled with an internal solution containing (in mM) the following: 130 K-gluconate, 10 KCl, 10 HEPES, 1 EGTA, 2 MgCl_2_ adjusted to pH 7.35 and osmolarity of 262 mOsm. For recording of DAT-mediated whole-cell currents, pipettes were filled with (in mM) the following: 120 CsCl, 30 dextrose, 10 HEPES, 1.1 EGTA, 2 MgCl_2_ and 0.1 CaCl_2_ adjusted to pH 7.35 and osmolarity of 264 mOsm. Recordings were performed at 37 °C. For determining DAT-mediated current and IV changes at different holding potentials, a stable IV (−100 to +40 in 20 mV steps) was generated after 5 min of continuously holding the cell at the given potential (−60, −40 or +20 mV), and then the DAT blocker GBR12935 (20 μM) was added to the bath and subsequent IVs were measured every 30–60 s. To determine the DAT-mediated current amplitude, the IV in the presence of GBR12935 (Fig. 6c; protocol #2; grey traces) was subtracted from the preGBR12935 IV following the prolonged clamp of the MP (Fig. 6c; protocol #1; red=+20 mV, black=−40 mV, blue=−60 mV) to yield the DAT-mediated (GBR12935-sensitive) current at each voltage step of the IV (the curve displayed in Fig. 6d) corresponding with manipulation of the MP state in previous TIRFM experiments (Fig. 5). For statistical comparisons between groups (Fig. 6e,f), the DAT-mediated current (pre-GBR12935–post-GBR12935 amplitude) at the −100 mV step, where DAT-mediated current is largest, was used.

For recording and imaging of DAergic neurons in primary culture, the external solution contained (in mM) the following: 146 NaCl, 5 HEPES, 5 KCl, 30 Dextrose, 2.5 CaCl_2_ and 1.2 MgCl_2_, with pH 7.35 and had an osmolarity of 290–300 mOsm. Patch pipettes (4–6 MΩ) were filled with an internal solution containing the following (in mM): 135 K-gluconate, 5 KCl, 2 MgCl_2_, 10 HEPES, 0.1 EGTA, 2 Mg-ATP and 0.2 Na-GTP, pH adjusted to 7.35 and osmolarity of 270 mOsm. Recordings of the neuronal MP were corrected offline for a calculated liquid junction potential of 16.1 mV. All recordings occured at 37 °C.

### Microscopy

All microscopy analyses were performed at 37 °C, and cells were washed twice with external solution as described above before all experiments. For all imaging experiments, cells/neurons were set on 35-mm glass-bottom dishes (MatTek, Ashland, MA) with glass thickness of 0.13–0.16 mm (TIRFM) or 0.085–0.13 mm (confocal). Wide-field fluorescence images were acquired identically to TIRFM images; however, a Lambda LS Xenon Arc Lamp provided the light source that bypassed the additional TIRFM mirror set and was passed through appropriate excitation (Ex)/emission (Em) filters and dichroic mirror. Microscopy data were analysed in the Nikon's NIS Elements software.

*TIRFM*. TIRFM imaging of HEK cells and neurons plated on poly-d-lysine-coated dishes was performed at 37 °C using Nikon Eclipse TE-2000-U inverted microscope, with a × 60 1.49 numerical aperture (NA) objective and equipped with a multiline solid-state laser system (470, 514 and 561 nm) and appropriate filter combination for YFP (Ex: 514 nm/Em: 535 nm), TagRFP (Ex: 561 nm/Em: 584 nm) and CFP (Ex: 445 nm/Em: 475 nm), similar to as previously described^57^. TIRFM was achieved via the ‘through-the-objective' laser guidance method with the laser incident angle set to 76°, which is greater than the critical angle of 62° and generated an evanescent field depth between 66 and 72 nm depending on the wavelength of light used. Temperature control was maintained with a stage and an objective heater (20/20 Technology Inc.). Image exposure time was coupled with laser excitation duration at 200–300 ms, and laser intensity was maintained at 40–60% of maximum intensity, but neither changed throughout the course of a given experiment. Images were detected digitally using an attached CoolSNAP HQ^2^ CCD camera and stored on a computer hard drive at 5–10-s intervals. For imaging of HEK cells, baseline images were acquired during perfusion of standard external solution before changing the solution to 100-mM KCl-based external solution (osmotically balanced) or 10 μM AMPH, prepared as described above, or throughout the entirety of being held in the whole-cell configuration. For simultaneous patch-clamp and TIRFM, membrane-holding potentials of +20, −40 and −60 mV were determined in preliminary experiments to approximate endogenous resting potential of these cells (−40 mV), to mimic the effects of 100-m KCl depolarization (+20 mV) and to oppose depolarizing effects of +20 mV with a similar magnitude of change (−60 mV).

For quantification of cell-surface fluorescence intensity of isolated HEK cells and primary culture neurons, regions of interest were created, including the TIRFM footprint of each HEK cell in its entirety or the neuron's soma. For all image sequences, a background ROI similar to the size of a cell was placed in a region devoid of cells/fluorescence and was subtracted from the entire image and recalculated for each frame. The mean intensity (in arbitrary fluorescent units) over time was monitored and plotted/analysed as a fraction of the baseline intensity (the mean raw intensity of all frames within 30–60s before initiation of indicated manipulation) and used for analysis. Bleaching was controlled for in two ways. The first was the inclusion of a vehicle group and/or non-patched adjacent cells for each assay appropriately. However, because of relatively large observed cell-to-cell variability in the change in baseline fluorescence over time ranging from −3.0 to +2.1% per min (average 0.4±0.3% per min), a correction factor or rate was determined for each cell and was used to account for this change in each cell. Since the bleaching rate with the current TIRFM imaging parameters was linear, a linear fit was generated for 120 s before a solution change and was used to determine that the rate of bleaching was extrapolated over the entire 12–15-min experiment. This projected rate of change in fluorescence intensity due to bleaching was then accounted for during each experiment.

*Confocal microscopy and JHC 1-064 labelling of DAT*. Imaging of YFP-DAT (ex: 514 nm, em: 540/30 nm), mCherry, dsRed and JHC 1-064 (all ex: 561 nm, em: 585/65 nm) was performed using the Nikon A1R confocal system mounted on a Nikon Eclipse Ti-E inverted microscope (Nikon) using a × 60 1.49 NA Plan-Apo objective (Nikon). For YFP-DAT and endosome co-localization experiments, YFP-DAT HEK cells grown on glass-bottom dishes and transfected with TagRFP-T-EEA1 or DsRed-Rab11 were treated with either 100 mM KCl for 5 min, 10 μM AMPH for 1 h or with standard external solution vehicle throughout all the experiments at 37 ° C. After the treatment, the dishes were placed on ice and washed with the ice-cold standard external solution, then washed two more times with ice-cold PBS solution and then fixed in 3.7% paraformaldehyde. Cells were then washed and imaged using identical imaging parameters (for example, laser power, gain, and so on) immediately in PBS. For co-localization analysis, a region of interest (ROI) was drawn over the intracellular space of each cell in the raw image and the Pearson correlation coefficient for the two channels was calculated on a cell-by-cell basis in NIS Elements (Nikon). For clarity and image display only, a single-count 3 × 3 pixel matrix smooth was applied, and intensity of all pixels was enhanced by 40%.

The fluorescent cocaine analogue, JHC 1-064, which has a high affinity for DAT, was used as previously described to selectively label membrane-resident DAT^35^,^36^,^38^,^66^. When YFP-DAT HEK cells had reached 60–80% confluency after 2–3 days or midbrain primary culture neurons had reached DIV 5, they were washed twice with the appropriate standard external solution and incubated with 10–20 nM JHC 1-064 for a minimum of 30 min at 4 °C. After at least 30 min, the JHC 1-064-containing solution was removed and replaced with fresh 4 °C external solution. Immediately, the dish was placed on the stage, and cells were selected and a baseline image was acquired. Following acquisition, the cold solution was removed and replaced with either vehicle or KCl-based external solution at 37 °C, and images were acquired every 5 min. Imaging parameters (for example, laser power, gain, pinhole, and so on) were identical for images of HEK cells and were within the imaging series of each neuron. For analysis of JHC 1-064/DAT complexes in HEK cells, an area devoid of cells was selected and used as a background ROI, and the mean pixel intensity of this region was subtracted from intensity of all pixels. For determination of the mean intracellular intensity, an ROI was drawn within the boundaries of each cell and the mean intensity (normalizing for changes in cell size) was again normalized to the intensity of the entire image to account for bleaching and was plotted as a fraction of the initial internal fluorescence in the original control 4 °C image^67^. For manual counts of intracellular JHC 1-064 puncta and clarity for display (Fig. 3 and Supplementary Fig. 8), all images were processed identically.

### Biotinylation assay

For biotinylation assays, YFP-DAT HEK cells or parental HEK293 cells were plated on 24-well poly-D-lysine-coated plates at a density of 2 × 10^5^ cells per well and transfected with either GFP-C1-CaMKIIα-K42R or pTfR-PAmCherry1, as previously described^68^. Forty-eight to ninety-six hours after plating, cells were pre-treated (30 min) with external solution (vehicle) or 80 μM Dynasore, followed by 30-min treatment with vehicle, vehicle+10 μM KN92 or vehicle+10 μMKN93. Cells were then washed and treated for 5 min with either vehicle, 10 μM AMPH, 2.5 μM PMA, iso-osmotic 50 mM KCl, iso-osmotic 100 mM KCl, 100 mM KCl+KN92 or 100 mM KCl+KN93. Cell-surface proteins were then biotinylated and analysed via western blot analysis as described previously^69^. Total protein concentrations for each sample were determined using the Pierce BCA protein assay kit (Thermo Scientific), and the resulting values were used to load equal amounts of protein for each sample when conducting SDS–PAGE. Blots of total and surface protein (Supplementary Figs 4c,d and 5c for original blots) were probed with an N-terminal-targeted anti-DAT monoclonal antibody (1:1,000; mAb16 (ref. 70); a gift from Dr Roxanne Vaughan of the University of North Dakota) or an anti-transferrin receptor antibody (1:1,000; C2F2, BD Biosciences), and the density of the immunoblot bands was quantitated using ImageJ (NIH) or Image Studio (LI-COR). Prism5 (GraphPad) was used for statistical analysis following normalization of surface values to vehicle and determination of the vehicle variance by normalizing to AMPH.

### Drug/solution application and optical stimulation

In electrophysiology, microscopy and biochemistry experiments, increased KCl concentrations (100 mM) were achieved by replacing NaCl in the standard external solution or ACSF with KCl in an equa-molar manner, conserving osmolarity. For TIRFM imaging of HEK cells, vehicle (standard external solution), the KCl-based external solution or 10 μM AMPH was applied via bath perfusion using a laminar flow insert for 35-mm Petri dishes at a rate of 2 ml min^−1^. For neuronal recordings and TIRF microscopy, vehicle or KCl-based external solution was applied via pressure (2 psi) injection from a pipette identical to recording pipettes positioned 20 μm from the cell body. HEK293 or YFP-DAT HEK cells were exposed to either 80 μM dynasore^34^ (Thermo Fisher Scientific), 10 μM KN92, 10 μM KN93 or 10 μM BIM (all from EMD Millipore) for 25–30 min before and throughout treatment with vehicle or 100 mM KCl to maintain the respective inhibition of dynamin, CaMK and PKC throughout each imaging or biotinylation experiment as indicated.

For steady-state photo-activation of eArch3.0 in cultured neurons, 590-nm light was generated from a light-emitting diode (LED) source (Thorlabs) and coupled to an optical fibre and placed at a 45 degree angle, with the tip 150–200 μm from the cell body. The output at the fibre tip (200 μm diameter) was regulated via a potentiometer on the externally-triggered LED driver and was calibrated so that the light power density at the tip was 15 mW mm^−2^. Changes in MP in response to eArch 3.0 activation were determined by taking the average MP over 50 ms before light onset and the last 50 ms of a 1-s light pulse.

### Data analysis

All data were analysed with Microsoft Excel, IBM SPSS, Prism5 or Igor Pro. Statistical analyses used for comparison are identified in the legend, and all values are the mean±s.e.m., unless otherwise stated.

## Additional information

**How to cite this article:** Richardson, B. D. *et al*. Membrane potential shapes regulation of dopamine transporter trafficking at the plasma membrane. *Nat. Commun.* 7:10423 doi: 10.1038/ncomms10423 (2016).

## Supplementary Material

Supplementary Information Supplementary Figures 1-8 and Supplementary Methods.

Supplementary Movie 1 Temporally compressed TIRFM imaging sequences of HEK YFP-DAT cells during the bath perfusion of depolarizing 100mM KCl. Note rapid loss of YFP-DAT signal on the basal surface and edges at 0:01sec and subsequently return at 0:03sec.

Supplementary Movie 2 Temporally compressed TIRFM imaging sequences of primary cultured neuron soma expressing RFP-DAT before and during application of depolarizing 100mM KCl. Note rapid loss of RFP-DAT signal on the basal surface beginning at 0:03sec.

Supplementary Movie 3 Temporally compressed TIRFM imaging sequences of primary cultured neuron soma expressing CFP-DAT before, during and after activation of archaerhodopsin with 590nm light between frames. Note steady increase in RFP-DAT signal beginning at 0:01sec and gradual reduction beginning at 0:03sec, in line with photo-stimulation duration.
