# Supplementary material for: Membrane potential shapes regulation of dopamine transporter trafficking at the plasma membrane
Source: Nat Commun. 2016 Jan 25;7:10423. doi: 10.1038/ncomms10423 (PMC4737753; doi:10.1038/ncomms10423)
Supplement: Supplementary Information — Supplementary Figures 1-8 and Supplementary Methods. [file ncomms10423-s1.pdf]

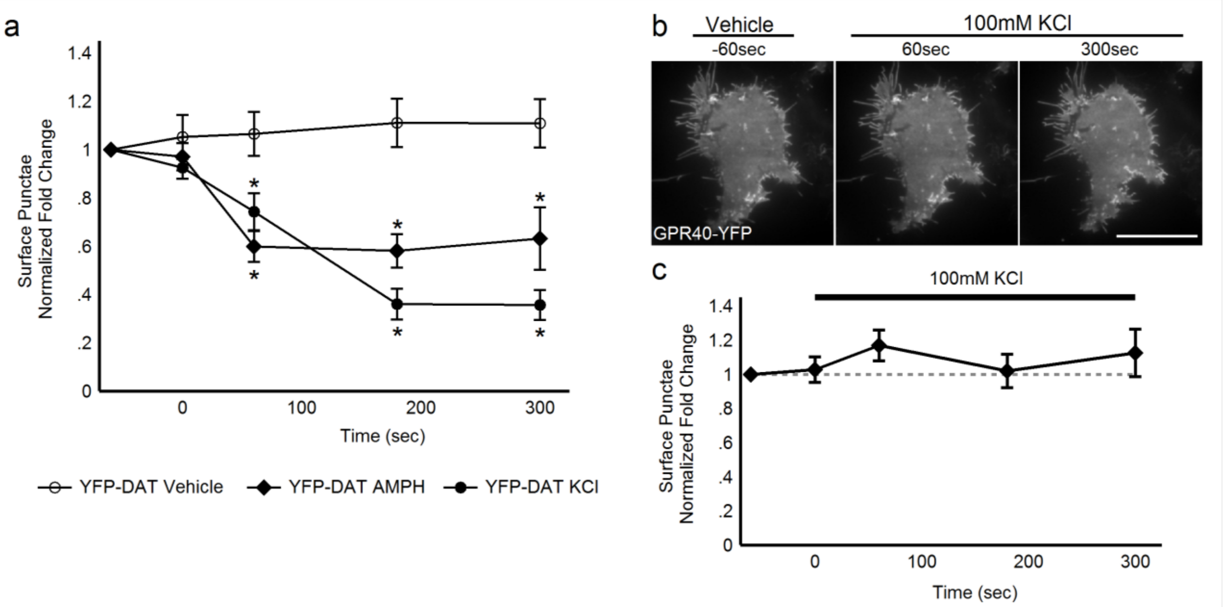

Supplementary Figure 1. Amphetamine causes redistribution in membrane DAT and the GPCR, GPR40, is insensitive to depolarization-induced changes in membrane expression. a) Timecourse plot of mean±SEM normalized (to pre-KCl time point) number of membrane surface-resident YFP-DAT punctae before and during vehicle (open circles; *N*=5, *n*=17), 10µM amphetamine (diamonds, *N*=5, *n*=14) or 100mM KCl (closed circles; *N*=6, *n*=14) bath application showing the amphetamine-induced loss of membrane DAT stabilizes after 2min and is not as dramatic and depolarization-induced membrane DAT loss. *$p<0.05$ compared to control with independent samples *t*-test. b) Distribution of the membrane G-protein coupled receptor, GPR40-eYFP, is unaffected by membrane depolarization. Live cell sequential TIRF microscopy images of GPR40-eYFP-transfected HEK cells 60sec before and throughout 5min perfusion with depolarizing 100mM KCl-based external solution (*N*=7, *n*=7). Scale bar is 20µm. c) Timecourse plot of mean±SEM normalized (to pre-KCl time point) number of membrane surface-resident GPR40-eYFP punctae before and during 100mM KCl bath application indicates the relative insensitivity of the trafficking of this naturally-occurring membrane protein to depolarization (*N*=7, *n*=7).

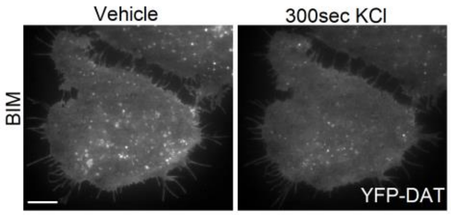

Supplementary Figure 2. Depolarization-dependent redistribution of YFP-DAT is not PKC-dependent. Representative TIRF microscopy images of HEK YFP-DAT cells 60sec before and throughout 5min perfusion with 100mM KCl pre-incubated with 10µM bisindolylmaleimide I (BIM; *N*=4, *n*=8). Scale bar is 10µm.

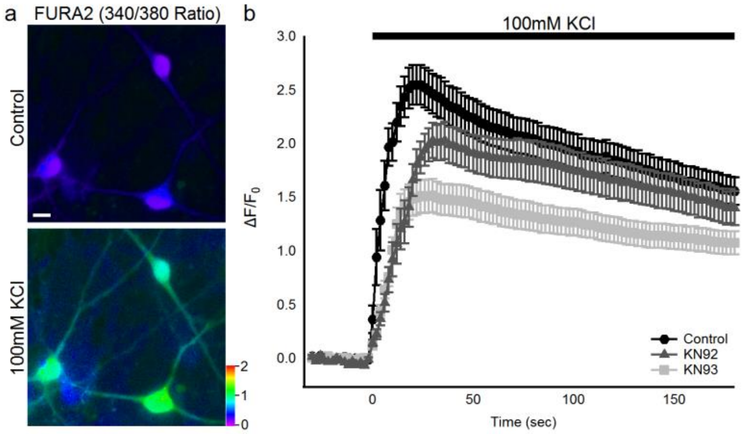

Supplementary Figure 3. CaMKII inhibitor, KN93, attenuates but does not abolish depolarization-induced Ca^{2+} influx. a) Representative Fura-2 ratiometric (340nm/380nm) image of midbrain primary culture neurons before (top) and during the peak Ca^{2+} signal (bottom) evoked with bath application of 100mM KCl in control conditions. Image color scale of raw ratio values for emission intensity excited by 340nm/380nm light. b) Mean±SEM normalized change in FURA2 fluorescence ($\Delta F/F_0$) ratiometric signal in response to 100mM KCl-based external solution (black; control) and when the CaMKII inhibitory, KN93 (10µM; light gray), or its inactive homolog, KN92 (10µM; dark gray), are pre-incubated with the neuronal cultures and included in the bathing solution. The administration of 100mM KCl-based external

solution is indicated by the black bar. Values are mean±SEM. *N*=4-5 experiments and *n*=23-26 cells/group.

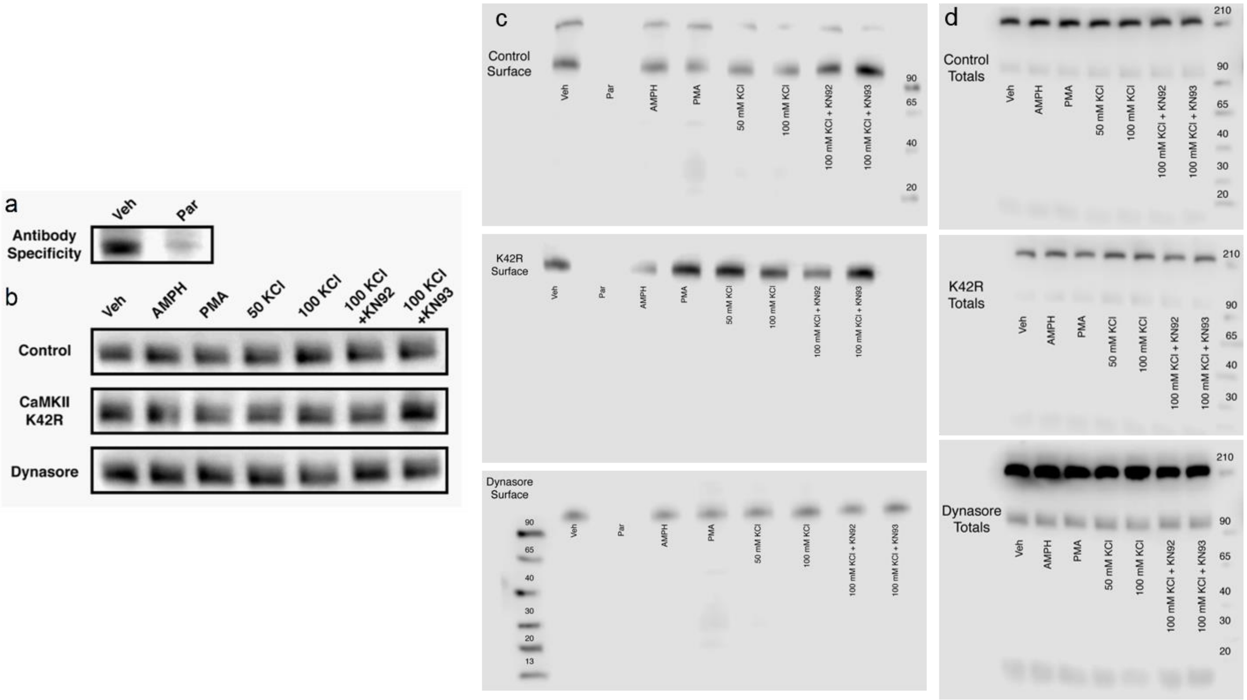

Supplementary Figure 4. Antibody specificity validation and total DAT representative Western blots for each condition corresponding to Figure 3. a) DAT antibody labeling in YFP-DAT HEK (left) and parental HEK (right) cell lysates. b) Representative total DAT protein blots for each condition. c-d) Example full blots for surface (biotinylated) DAT (c) and total DAT (d) in for all conditions.

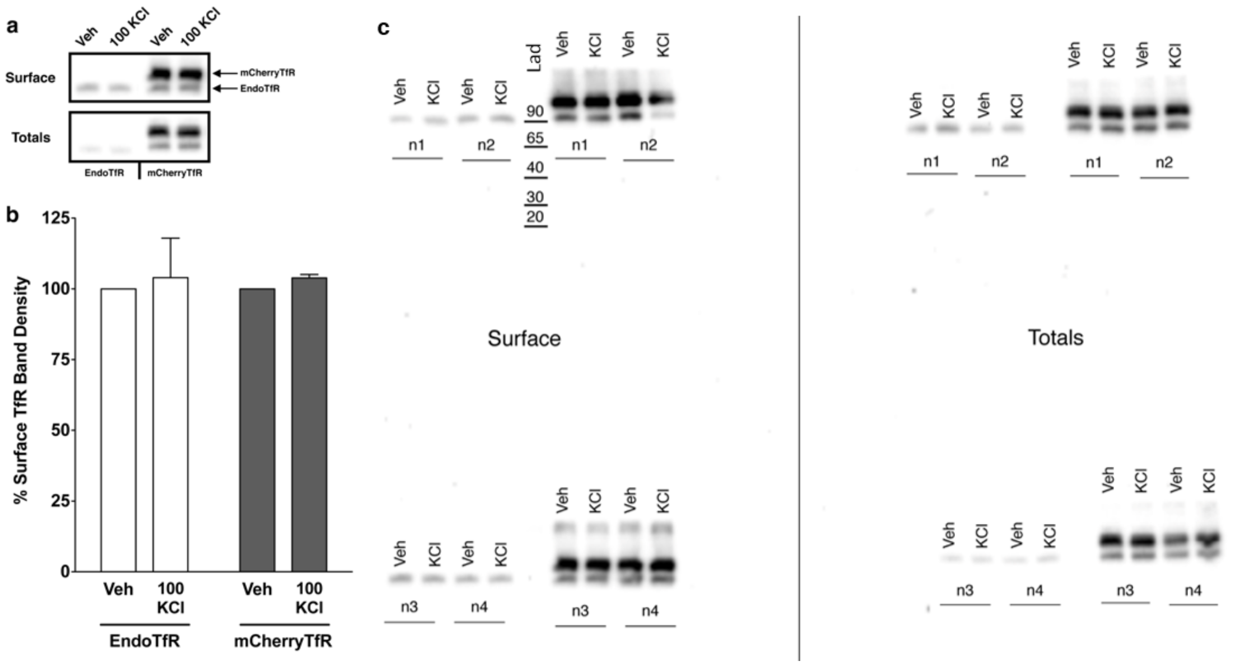

Supplementary Figure 5. Membrane levels of the endogenous transferrin receptor (TfR) are unchanged upon membrane depolarization. (a) Representative Western blots show the surface and total TfR present when HEK-293 cells (left, EndoTfR) or HEK-293 cells transfected with a positive control pTfR-PAmCherry1 plasmid (right, mCherryTfR) were treated for five minutes with either standard external solution (veh) or 100mM KCl-based external solution. (b) Mean±SEM surface TfR band density from HEK-293 cells when normalized to vehicle treatment (*n*=3) indicate that surface levels of endogenous TfR or mCherry-tagged TfR are unchanged upon membrane depolarization (*n*=3). Arrows indicate endogenous and mCherry TfR as labeled. c) Combination of whole blots of surface (biotinylated) and total TfR used to generate a and b. Endogenous and mCherryTfR were considered separately and differences were determined using paired *t*-tests.

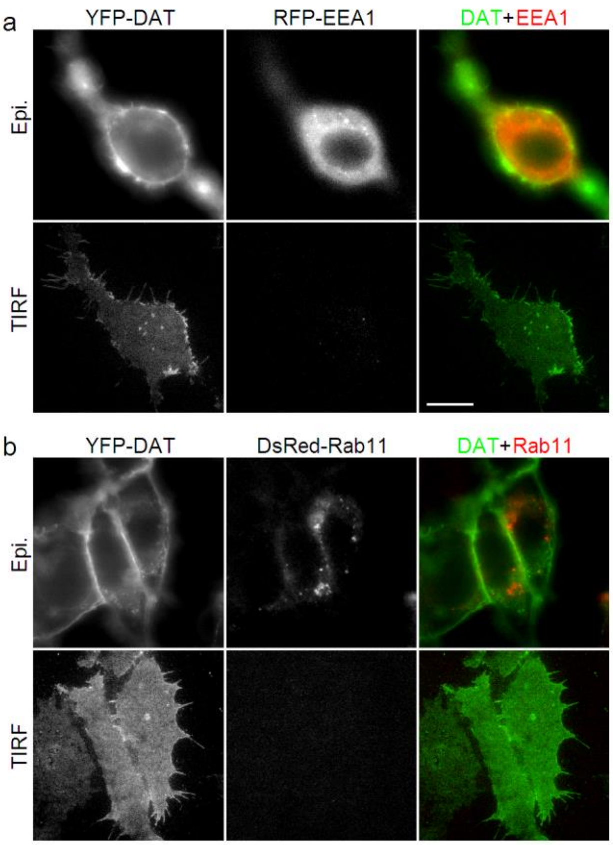

Supplementary Figure 6. Endosome markers, EEA1 and Rab11, are not visible at the surface membrane in the TIRF footprint. Representative images of HEK YFP-DAT cells expressing RFP-EEA1 (a) or DsRed-Rab11 (b) in epifluorescence (top) or TIRF (bottom). Intracellular endosome markers are present in epifluorescence and confocal (Figure 4), but not detectable above background in TIRFM, suggesting they are not present at the membrane and confirmation that fluorescence signal in TIRF is largely from membrane-resident proteins.

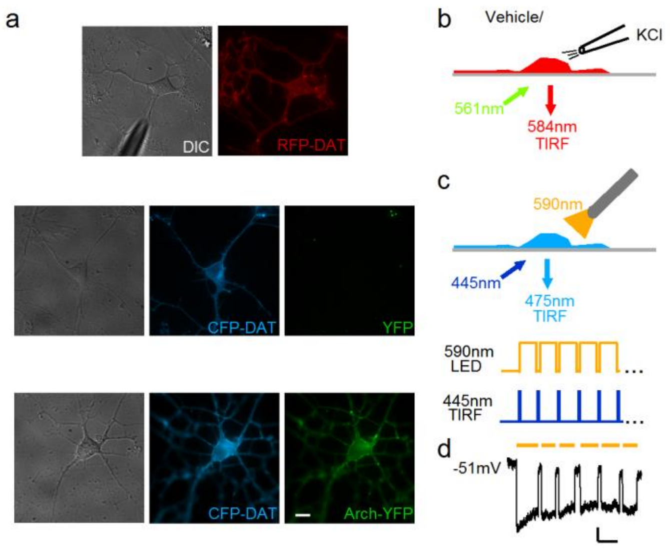

Supplementary Figure 7. Fluorescently-tagged protein expression and imaging scheme for TIRF imaging of neurons and example effect of resulting prolonged Arch activation. a) DIC and fluorescent images of midbrain primary culture neurons expressing TagRFP-T-DAT with picospritzer pipette positioned 20µm from soma (top), CFP-DAT (middle) or CFP-DAT and Arch-YFP (bottom) imaged in wide-field fluorescence. b-c) Schematic of simultaneous TIRF imaging of TagRFP-T-DAT and transient KCl-induced (applied via picospritzer pipette) membrane depolarization (b) or TIRF imaging of CFP-DAT and transient Arch-activation-induced membrane hyperpolarization via stimulation with adjacent optical fiber placed near the soma (c, upper). c) Lower: For imaging, the timing of Arch photostimulation was continuous except during the 200-300ms TIRF illumination and exposure period during which time the 590nm LED light source was inactive. d) Representative recording of membrane potential from primary culture neuron expressing Arch-YFP in response to pulses of 590nm light stimulation via adjacent optical fiber corresponding to temporal parameters of stimulation/pauses during simultaneous TIRF imaging. Scale bar is 5sec and 5mV.

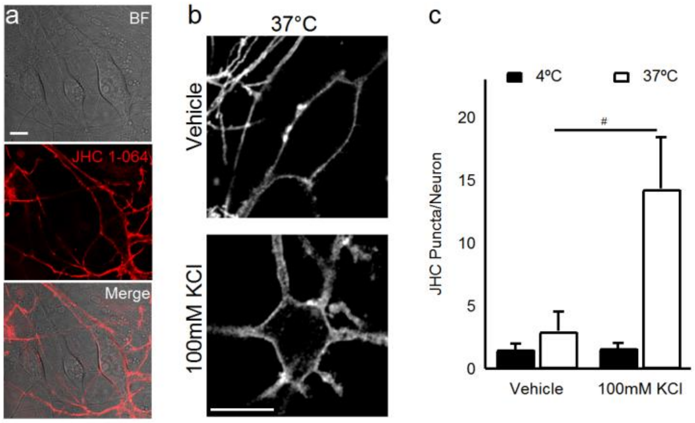

Supplementary Figure 8. Depolarization enhances internalization of JHC1-064/DAT complexes in midbrain primary cultured neurons. a) JHC 1-064 (10nM for 30min) labels membrane DAT in a subset of mouse midbrain primary culture neurons. Scale bar is 10µM. b) Single plane confocal image of primary neuron cell bodies labeled with JHC 1-064 imaged 5min after replacing bath solution with 37°C standard (vehicle; top) or KCl-based external solution (bottom). Note JHC 1-064 punctae present only in the intracellular space of the neuron exposed to depolarizing KCl-based external solution. Scale bar is 20µM. c) Average number of JHC 1-064 punctae in the intracellular space of neurons at 4°C and after being at 37°C in standard (n=5) and KCl-based (n=3) external solution. Within a given condition, the same cells are represented in the 4°C and 37°C states. #$p<0.05$ using independent samples t-test. Values are mean±SEM.

Supplementary Methods

Neuronal intracellular free calcium $[Ca^{2+}]_i$ was measured using cell-permeant, ratiometric calcium dye Fura-2 AM (Life Technologies). Primary culture of midbrain neurons were washed twice with 37^oC external solution supplemented with 1% bovine serum albumin (fatty acid free). Neurons were then incubated in external solution containing 5µM Fura-2AM in dark at 37^oC for 40min. Cultures were then washed twice with supplemented external solution and incubated at 37^oC for another 20min in either external solution (control) or external solution containing KN93 (10µM) or KN92 (10µM). KN93 or KN92 were also present in the bathing solution throughout subsequent imaging. The neurons were then incubated for another 10min under continuous perfusion of standard external before switching the bath perfusion to 100mM KCl-based external, all at 37^oC on an inverted Nikon TE2000E microscope, before starting the imaging using a CoolSNAPHQ$_2$ camera and 40x, 1.3 oil immersion Plan Fluor objective lens. The Fura-2 dye was excited using excitation wavelengths of 340nm (for Ca bound Fura-2) and 380 nm (for free Fura-2) and emission detected at single wavelength of 510nm with images collected at 1Hz. Both image acquisition and analysis were done using Nikon Elements Advanced Research imaging software (Nikon Instruments) and data was quantified in a ratiometric manner and normalized to basal values.
